# Examination of Different Accelerometer Cut-Points for Assessing Sedentary Behaviors in Children

**DOI:** 10.1371/journal.pone.0090630

**Published:** 2014-04-03

**Authors:** Youngwon Kim, Jung-Min Lee, Bradley P. Peters, Glenn A. Gaesser, Gregory J. Welk

**Affiliations:** 1 Department of Kinesiology, Iowa State University, Ames, Iowa, United States of America; 2 School of Health, Physical Education and Recreation College of Education, University of Nebraska, Omaha, Nebraska, United States of America; 3 School of Nutrition and Health Promotion, Arizona State University, Phoenix, Arizona, United States of America; University of Massachusetts, United States of America

## Abstract

**Background:**

Public health research on sedentary behavior (SB) in youth has heavily relied on accelerometers. However, it has been limited by the lack of consensus on the most accurate accelerometer cut-points as well as by unknown effects caused by accelerometer position (wrist vs. hip) and output (single axis vs. multiple axes). The present study systematically evaluates classification accuracy of different Actigraph cut-points for classifying SB using hip and wrist-worn monitors and establishes new cut-points to enable use of the 3-dimensional vector magnitude data (for both hip and wrist placement).

**Methods:**

A total of 125 children ages 7–13 yrs performed 12 randomly selected activities (from a set of 24 different activities) for 5 min each while wearing tri-axial Actigraph accelerometers on both the hip and wrist. The accelerometer data were categorized as either sedentary or non-sedentary minutes using six previously studied cut-points: 100counts-per-minute (CPM), 200CPM, 300CPM, 500CPM, 800CPM and 1100CPM. Classification accuracy was evaluated with Cohen's Kappa (κ) and new cut-points were identified from Receiver Operating Characteristic (ROC).

**Results:**

Of the six cut-points, the 100CPM value yielded the highest classification accuracy (κ = 0.81) for hip placement. For wrist placement, all of the cut-points produced low classification accuracy (ranges of κ from 0.44 to 0.67). Optimal sedentary cut-points derived from ROC were 554.3CPM (ROC-AUC of 0.99) for vector magnitude for hip, 1756CPM (ROC-AUC of 0.94) for vertical axis for wrist, and 3958.3CPM (ROC-AUC of 0.93) for vector magnitude for wrist placement.

**Conclusions:**

The 100CPM was supported for use with vertical axis for hip placement, but not for wrist placement. The ROC-derived cut-points can be used to classify youth SB with the wrist and with vector magnitude data.

## Introduction

Excessive time spent on sedentary behavior (SB) in youth is associated with metabolic riskfactors [1], cardiorespiratory fitness [2], childhood obesity [3], and mental health [4].This is a significant public health concern considering that time spent in SB has been shown to be increasingin all age [5] and ethnicity/socioeconomic groups [6]. To advance research on SB it is critical to obtain precise estimates of time youth spend being sedentary.

Accelerometry-based activity monitors such as the Actigraph (Actigraph LLC, Pensacola, FL, USA) have been widely used to assess time spent in physical activity, but there is no consensus on the most accurate “cut-point” to classify time spent in SB. The ideal cut-point for SB would accurately distinguish sedentary activities from physical activities but many different values have been proposed and/or derived from previous studies. The value of 100 counts per minute (CPM) [7]–[9] has been used in the National Health and Nutrition Examination Survey (NHANES) [10], [11], as well as in other surveillance [12]–[14] and health outcome [15], [16] studies. However, other cut-points have been used for identifying correlates of SB (i.e. 200CPM [17] and 1100CPM [18]), tracking behavior patterns (i.e. 800CPM [19]) and examining heath impacts of SB (i.e. 800CPM [20] and 500CPM [21], [22]). Still other SB cut-point values have been proposed and/or derived in other published studies: 41CPM [23], 288CPM [24], 372CPM [25], and 420CPM [26].

The use of different cut-points has led to disparate estimates of SB, making it difficult to understand health outcomes of SB in youth. In fact, a recent study [27]demonstrated that associations between SB and metabolic risk factors were moderated by the choice of cut-point, with stronger associations being observed with higher cut-points (1100CPM) than with the more commonly used 100CPM value. Another study [28] also found out that relationships of SB with various adiposity and metabolic risk indicators varied when different sets of sedentary cut-points were applied (i.e. 420CPM [26], 288CPM [24] and 41CPM [23]). The complexities of determining optimal cut-points are further complicated by options for accelerometer placement and accelerometer output. TheNHANES has elected to collect physical activity (and SB) data with Actigraph accelerometers being placed on the wrist; however, it is premature to assume that cut-points developed for the hip can be used for wrist-worn monitors. The literature on Actigraph SB cut-points was based on uni-axial output and this provides little insight about how to interpret three-dimensional vector magnitude data.

The present study fills these gaps by 1) evaluating classification accuracy of six different vertical axis sedentary cut-points (100, 200, 300, 500, 800 and 1100CPM), 2) comparing classification accuracy between the hip- and wrist-worn monitors for the same activity, and 3) identifying a new set of sedentary cut-points to enable researchers to use vertical axis counts or vector magnitude measured from the Actigraph being placed on the hip and wrist.

## Materials and Methods

### Study Protocol

A total of 125 children aged 7–13 yrs participated in the present study. Physical characteristics of the participants are summarized in Table 1. Each participant performed a set of 12 activities randomly chosen from a pool of 24 activities of varying intensity: 5 sedentary, 3 light, 11 moderate and 5 vigorous activities (See Table 2). These criterion intensities of activities were defined a-priori on the basis of predicted METs from the Compendium of Physical Activities [29]: 1.0<MET≤1.5 for SB, 1.5<MET≤3.0 for light, 3.0<MET≤6.0 for moderate and 6.0<MET for vigorous intensity.Each activity was performed for 5 min, with a 1-min resting interval between different activities. All activities were performed in a supervised research setting. Because the type and timing of sedentary activities in the protocol were directly observed and tracked, we have an objective criterion measure of SB to evaluate the different sedentary cut-points. The data were collected as an ancillary study of a larger calibration and cross validation project funded by the National Institutes of Health. The final dataset included records from 125 children (76 with a hip-worn GT3X Actigraph and 49 with both hip-worn and wrist-worn GT3X+ Actigraphs).

**Table 1:** Descriptive statistics of the included participants.

*Anthropometric Measures*	All (n = 125)	Wrist only (n = 49)
**Age (yrs)**	9.9±1.9	10.1±2.1
**Gender**		
Female; n (%)	53 (42.4%)	29 (59.2%)
Male; n (%)	72 (57.6%)	20 (40.8%)
**Height (cm)**	145.7	147.3
**Weight (kg)**	38.2	38.6
**Body Mass Index (BMI)**	17.5±3.5	23.2±1.2
Normal Weight; n (%)	104 (83.1%)	43 (87.8%)
Overweight; n (%)	13 (10.4%)	2 (4.1%)
Obese; n (%)	8 (6.4%)	4 (8.2%)

**Table 2:** Descriptions of the included activities by intensity.

Intensity	Activity Type	Description
Sedentary	Sitting on a chair	Sitting on a chair quietly
	Reading a book	Reading a book/magazine selected by a researcher while seating on a chair at a desk.
	Watching TV	Watching a TV show/movie selected by a researcher while seating on a chair at a desk.
	Typing at a computer	Typing a magazine sitting on a chair at a laptop computer
	Playing a video game	While sitting on a chair desk, playing a video game provided by a researcher
Light	Very slow walking	Walking at a self-selected speed (encouraged to walk slower than his/her normal walking speed) on wood floor in a gym
	Loading and unloading boxes	Moving 3 boxes stacked on the top of a chair to another chair one at a time
	Playing catch	Throwing and catching a ball with a partner from a 15–20 ft distance
Moderate	Passing a basketball	Passing and receiving a basketball with a partner maintaining 15–20 ft
	Sweeping	Sweeping shredded pieces of paper on wood floor in a gym with a broom
	Walking at casual pace	Walking at a self-selected speed (encouraged to walk at his/her normal walking speed) on wood floor in a gym
	Brisk walking	Walking at a self-selected speed (encouraged to walk faster than his/her normal walking speed) on wood floor in a gym
	Hand weight exercises	Lifting a 5–10 kg (decided by a researcher's visual inspection on a participant's physical maturation) dumbbell up and down constantly
	Shooting a basketball	Standing in the free throw zone on a regular size basketball court, shooting a basketball towards the rim
	Walking, including stair climbing	Starting at a marked point 20 ft away from a stack of 3 staircases, walking to and climbing up and down the staircases.
	Stationary cycling at moderate pace	Cycling a bicycle ergometer at a self-selected speed (encouraged to ride at his/her normal biking speed)
	Dribbling a basketball	Walking dribbling a basketball around a rectangular marked with four cones
	Dribbling a soccer ball	Walking dribbling a soccer ball around the rectangular
	Light Calisthenics	Alternating between lunges (10 steps) and jumping jacks (10 times), with a 30 s break between them
Vigorous	Jogging at slow pace	Running lightly (encouraged to run at his/her normal running speed) around the rectangular
	Jogging at fast pace	Running fast (encouraged to run faster than his/her normal running speed) around the rectangular
	One-on-one basketball	Playing a one-on-one basketball game with a partner
	Stair climbing/stepping routine	Continuously stepping up and down on the 3 staircases
	Stationary cycling at vigorous pace	Cycling a bicycle ergometer at a self-selected speed (encouraged to ride faster than his/her normal biking speed)

### Ethics Statement

An assent form and written informed consent were signed by the children and their parents, respectively.This project was approved by the Institutional Review Board of Iowa State University.

### Accelerometer

The Actigraph GT3X (27 g; 3.8 cm×3.7 cm×1.8 cm) and GT3X+ (19 g; 4.6 cm×3.3 cm×1.5 cm) are light and small trial-axis accelerometers. They both yield activity counts for each of the 3 axes (vertical (x), anterior-posterior (y), medio-lateral axis (z)) and a value of vector magnitude (i.e. a square root of the sum of the x, y and z). The GT3X and GT3X+ use the same algorithms and filters (B. Matt, Actigraph LLC., personal communication, 2013), which allows for direct comparisons of outputs from the two devices. The GT3X and GT3X+s were initialized at 30 Hz and data were downloaded at 1 s epoch, then reintegrated into 60 s epoch. The low-frequency extension option in the Actigraph offers potential for detection of low intensity activities but it was not available for this study. Data were managed with the ActiLife software (firmware version 6.5.1).

### Statistical Analyses

Means and standard deviations of vertical axis counts and vector magnitudes for both hip and wrist placement were calculated. The six sedentary cut-points (100, 200, 300, 500, 800 and 1100CPM) for vertical axis were applied to the data to create a dichotomous categorization for every minute of the protocol: sedentary or non-sedentary. Receiver Operator Characteristic (ROC) curve analysis was used to identify optimal cut-points that best classified sedentary activities from non-sedentary activities for both the vertical axis (for the sake of comparison with the other six cut-points) and the vector magnitude for both the hip and wrist. The ROC-derived values were compared with the observed criterion categorization codes using agreement, Kappa statistics (Cohen's κ), sensitivity, specificity, positive predictive value (PPV), and negative predictive value (NPV). The benchmarks for Cohen's κ were “no agreement” (κ≤0), “slight” (0<κ≤.20), “fair” (.20<κ≤.40), “moderate” (.40<κ≤.60), “substantial” (.60<κ≤.80), and “almost perfect” agreement (.80<κ≤1.00) [30]. Receiver Operating Characteristic-Area Under the Curve (ROC-AUC) was used to identify optimal cut-points that best classified sedentary activities from non-sedentary activities for both the vertical axis (for the sake of comparison with the other six cut-points) and the vector magnitude for both the hip and wrist. Test of equality of ROC-AUC was performed to evaluate the agreement between the most accurate vertical cut-point (out of the six cut-points) and the optimal cut-point derived from the ROC-AUC analysis for hip placement. All of the analyses were performed using STATA/SE Version 12 for Windows (StataCorp LP, College Station, TX).

## Results

Vertical activity counts and vector magnitudes for the hip and wrist are presented in Table 3. The number of participants differed depending upon activity type due to the random selection of activities for each participant. Overall, for both the hip and wrist, the means and standard deviations of vertical axis counts and vector magnitudes were larger with higher intensities of physical activity. The means and standard deviations of vertical axis counts and vector magnitudes were also substantially larger for the wrist in comparison with the hip for 22 of the 24 activities performed. The only exceptions were for ‘stationary cycling at moderate pace’ and ‘stationary cycling at vigorous pace’.

**Table 3:** Activity counts and vector magnitudes for hip and wrist placement across 24 activities.

Intensity	Activity Type	Hip						Wrist					
		Vertical axis count			Vector Magnitude			Vertical axis count			Vector Magnitude		
		n	M	SD	n	M	SD	n	M	SD	n	M	SD
Sedentary	Sitting on a chair	67	31.6	79.6	67	118.1	219.7	27	390.8	682.7	27	899.5	1503.7
	Reading a book	64	22.5	86.3	64	77.5	207.9	21	373.5	630.5	21	1056.9	1208.8
	Watching TV	64	17.3	68.3	64	73.7	202.3	24	220.3	438.4	24	577.6	854.3
	Typing at a computer	72	18.7	73.3	72	112.7	237.6	33	350.4	377.9	33	1004.5	759.7
	Playing a video game	73	17.7	71.9	73	79.8	251.6	32	1253.6	1151.3	32	2518.0	1817.6
	***Total***	125	21.5	76.2	125	92.7	226.1	49	552.6	827.7	49	1275.4	1485.3
Light	Very slow walking	71	1344.6	835.1	71	2684.3	1023.1	26	2608.6	1406.8	26	4280.6	1945.6
	Loading and unloading boxes	81	1117.2	585.2	81	3188.6	919.7	27	5159.2	1276.8	27	7848.9	1806.0
	Playing catch	53	1139.5	820.6	53	3026.8	1533.9	19	10782.7	4375.9	19	17285.6	5941.3
	***Total***	115	1200.8	748.5	115	2973.8	1163.7	42	5748.5	4105.3	42	9092.6	6229.8
Moderate	Passing a basketball	47	707.5	776.1	47	2079.0	1343.1	29	13051.9	4468.3	29	18413.4	5368.8
	Sweeping	44	611.5	507.7	44	2843.4	1085.6	17	4908.0	1538.7	17	8215.3	2603.2
	Walking at casual pace	41	2421.8	888.2	41	3643.7	922.1	15	3876.3	1424.4	15	5780.2	2109.3
	Brisk walking	45	3421.5	1173.5	45	4714.6	1444.0	16	5040.5	2060.9	16	8518.3	3636.8
	Hand weight exercises	52	397.5	468.5	52	1089.7	778.9	27	7992.9	3236.4	27	11152.4	3967.5
	Shooting a basketball	13	3442.6	1923.0	13	5280.4	2360.6	0	-	-	0	-	-
	Walking, including stair climbing	38	2152.2	813.2	38	3849.2	1357.3	16	4157.1	1549.4	16	6411.9	2208.6
	Stationary cycling at moderate pace	60	788.5	1357.4	60	2518.0	1676.5	25	832.9	744.2	25	1435.3	1165.1
	Dribbling a basketball	58	2848.2	1287.4	58	4453.3	1644.8	20	14633.7	4230.0	20	20444.3	4958.8
	Dribbling a soccer ball	55	3135.3	1616.6	55	5152.1	1855.4	23	8174.8	4136.9	23	13443.6	6026.0
	Light Calisthenics	48	2797.9	2897.2	48	4343.2	3122.8	21	4778.9	4251.9	21	9340.2	7958.7
	***Total***	125	1955.2	1793.9	125	3504.2	2110.7	49	7130.1	5339.2	49	10796.7	7342.6
Vigorous	Jogging at slow pace	89	5177.1	1816.8	89	6316.9	1952.5	36	11467.7	4723.4	36	16854.1	6500.4
	Jogging at fast pace	100	5807.7	1987.1	100	7207.9	2129.5	39	14177.1	5440.9	39	20411.3	7317.9
	One-on-one basketball	38	2422.8	1539.9	38	4260.6	2272.7	7	11473.0	4534.5	7	17017.5	4670.9
	Stair climbing/stepping routine	106	2369.9	848.5	106	4465.5	1194.6	41	4332.6	1623.6	41	6902.6	2399.0
	Stationary cycling at vigorous pace	93	894.5	1264.7	93	2598.8	1723.3	36	1303.7	1610.6	36	2105.7	2419.9
	***Total***	125	3429.9	2467.5	125	5056.1	2492.5	49	7933.1	6384.8	49	11743.2	8904.9

M – mean; SD – standard deviation.

Table 4 summarizes statistics for classification accuracy of the six different sedentary cut-points for the hip and wrist. For the hip, the cut-point of 100CPM revealed high agreement (92.8%), “almost perfect” classification accuracy (i.e. Cohen's κ of 0.81), high sensitivity (93.7%), specificity (92.5%), PPV (78.8%) and NPV (98.0%). The statistics for 100CPM were slightly better than those for the other five cut-point values. For the wrist, none of the six cut-points yielded reasonably high statistics.

**Table 4:** Comparisons of different sedentary cut-points of the vertical axis for identifying sedentary activities.

Cut-point	Agreement (%)		Cohen's kappa (κ)		Sensitivity (%)		Specificity (%)		PPV^a^(%)		NPV^b^(%)	
	Point estimate	95%CI^c^	Point estimate	95%CI	Point estimate	95%CI	Point estimate	95%CI	Point estimate	95%CI	Point estimate	95%CI
***Hip (n = 125)***												
≤100	92.8	92.1, 93.3	0.81	0.79, 0.82	93.7	93.2, 94.3	92.5	91.9, 93.1	78.8	77.8, 79.7	98.0	97.7, 98.3
≤200	90.2	89.5, 90.9	0.76	0.74, 0.77	97.4	97.1, 97.8	88.1	87.3, 88.8	70.9	69.9, 72.0	99.1	98.9, 99.3
≤300	87.6	86.8, 88.3	0.70	0.68, 0.72	98.5	98.2, 98.8	84.4	83.5, 85.2	65.3	64.2, 66.3	99.5	99.3, 99.6
≤500	83.2	82.3, 84.0	0.62	0.59, 0.63	99.3	99.1, 99.5	78.4	77.5, 79.3	57.8	56.7, 58.9	99.7	99.6, 99.9
≤800	77.5	76.5, 78.4	0.53	0.48, 0.52	99.9	99.8, 99.9	70.8	69.8, 71.8	50.5	49.4, 51.7	100.0	99.9, 100.0
≤1100	73.2	72.1, 74.2	0.46	0.40, 0.44	100.0	100.0,100.0	65.2	64.1, 66.2	46.1	45.0, 47.3	100.0	100.0, 100.0
***Wrist (n = 49)***												
≤100	83.9	82.6, 85.3	0.44	0.38, 0.47	37.5	35.7, 39.2	98.2	97.8, 98.7	86.8	85.6, 88.0	83.6	92.3, 85.0
≤200	85.5	84.2, 86.8	0.53	0.48, 0.56	47.3	45.5, 49.1	97.3	96.7, 97.9	84.1	92.8, 85.4	85.7	84.5, 87.0
≤300	86.4	85.1, 87.7	0.58	0.54, 0.61	55.9	54.1, 57.7	95.8	95.1, 96.5	80.4	79.0, 81.9	87.6	86.4, 88.8
≤500	86.9	85.7, 88.2	0.62	0.58, 0.65	65.7	64.0, 67.5	93.5	92.6, 94.4	75.6	74.0, 77.2	89.9	88.8, 91.0
≤800	87.1	85.8, 88.3	0.65	0.62, 0.68	75.8	74.3, 77.4	90.6	89.5, 91.6	71.2	69.5, 72.0	92.4	91.5, 93.4
≤1100	86.9	85.7, 88.2	0.67	0.63, 0.69	84.0	82.7, 85.4	87.9	86.7, 89.1	68.1	66.4, 69.8	94.7	93.9, 95.5

a Positive Predictive Value;

b Negative Predictive Value;

c Confidence Interval.

From ROC-curve analyses, a new set of cut-points were derived for use with vertical axis and vector magnitude data from both the hip and wrist (See Table 5). The optimal cut-point identified for vertical axis for hip placement was 124CPM and this was determined to be statistically equivalent to the values with 100 CPM using a test of equality (Chi-square: 0.54, p-value: 0.4638). The optimal cut-point for vector magnitude for hip placement was considerably higher (554.3) but the diagnostic criteria were similarly strong. For wrist placement, the optimal cut-points for vertical axis and vector magnitude were 1756 and 3958.3, respectively. The corresponding diagnostic criteria values were also acceptably high. However, the values were slightly smaller than those for hip placement; in particular, the specificity values for wrist placement (83.5% for vertical axis and 79.7% for vector magnitude) were substantially smaller than those for hip placement (91.3% for vertical axis and 95.4% for vector magnitude).

**Table 5:** Identification of sedentary cut-points for the hip and wrist.

Comparison	Cut-point	ROC-AUC^a^ (95%CI)	Sensitivity % (95%CI)	Specificity % (95%CI)
***Hip (n = 125)***				
Vertical Axis	≤124	0.98 (0.97, 0.98)	95.1 (93.9, 96.1)	91.3 (90.6, 92.1)
Vector Magnitude	≤554.3	0.99 (0.98, 0.99)	96.5 (95.5, 97.3)	95.4 (94.8, 95.9)
***Wrist (n = 49)***				
Vertical Axis	≤1756	0.94 (0.93, 0.95)	93.0 (90.8, 94.8)	83.5 (81.9, 85.0)
Vector Magnitude	≤3958.3	0.93 (0.92, 0.94)	94.3 (92.3, 95.9)	79.7 (78.0, 81.4)

a Receiver Operating Characteristic-Area Under the Curve.

## Discussion

The present study systematically evaluated the validity of the six most commonly proposed Actigraph cut-points for classifying youth SB and investigated whether or not these cut-points could be used for the wrist. In addition, optimal cut-point values for vertical axis and vector magnitude were developed for both hip and wrist placement. The 100CPM cut-point with vertical axis data yielded the highest classification accuracy of the 6 values when worn on the hip. However, when the 100 CPM value was applied to vertical axis data collected from the wrist, substantively lower classification accuracy was obtained. The ROC-curve analyses supported the 100 CPM value of 100 for vertical axis data obtained from the hip since no significant differences were noted with the empirically derived value of 124 CPM. The other ROC-derived cut-points were highly variable (554.3CPM for vector magnitude at hip, 1756CPM for vertical axis at wrist, and 3958.3CPM for vector magnitude at wrist), demonstrating that the axis and location must be considered in SB monitoring studies.

The results for the vertical axis with the hip-worn Actigraph are consistent with other validation studies [31]–[33] that have supported the use of a 100CPM cut-point. A study by Ridgers et al. [32] directly compared various cut-points (50, 100, 150CPM) for classifying SB against data from an activPALin 48 children ages 8–12 yrs. In that study [32], the 100CPM produced estimates of sitting time most comparable with sitting time measured with the activPAL for school hours. Another well-designed validation study by Trost et al.[31] also confirmed the high classification accuracy of 100CPM for assessing SB in 206 children ages 5–15 yrs. In their study, the 100CPM value yielded substantially better classification accuracy (ROC-AUC: 0.90, sensitivity: 100%, specificity: 79.0%) in comparison to 800CPM (ROC-AUC: 0.80, sensitivity: 100%, specificity: 60.7%) in relation to indirect calorimetry data. Fischer and Yildirim et al.[33] also found the 100CPM value to yield better classification than the 300, 800, 1100CPM values using a similar reference methodology as the present study in a sample of 29 children ages 5–11 yrs. The convergence of findings indicates that the100CPM value is the best choice for assessing SB using the vertical axis data from an Actigraph worn at the hip.

An important and unique finding of the present study is that cut-points tested with hip-worn monitors cannot be applied for wrist-worn monitors. We observed large differences in activity counts and vector magnitudes for SB with monitors worn on the hip and wrist (see Table 3).These substantive differences between the hip and wrist are likely attributable to the generic characteristics of youth. To be specific, most SBs in youth typically involve large amounts of hand movement, while nearly no movements are likely to occur at the hip. For example, when reading a book and typing at a computer on a chair, it would be essential for a child to use their hands in order to flip through the pages of the book and enter text to the computer, respectively, both of which accordingly entail some movements of their wrist. For the same activities, however, almost no movement would be occurring at the hip if the person is seated on a chair. Thus, it is clear that cut-points developed and tested for hip-worn monitors cannot be applied to assess SB with wrist-worn monitors. Similarly, it is not possible to use previously developed cut-points with vector magnitude values.

**Figure 1:**
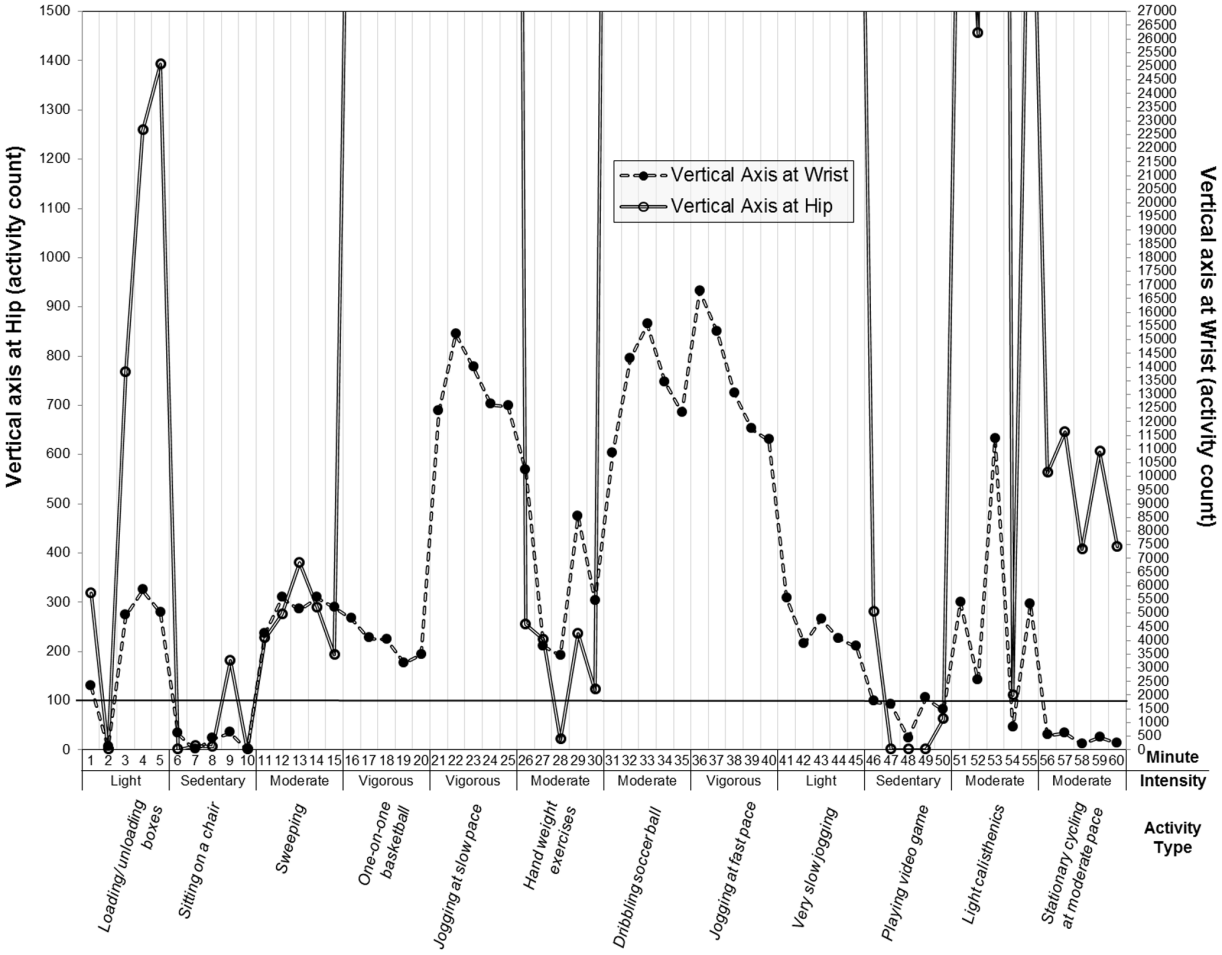
Illustration of the effects of the identified optimal cut-points for the vertical axis of the Actigraph being placed at the hip and wrist for a 9-yr old female participant. Note: the horizontal solid line specifies the cut-points for both the hip (100 counts per min; left Y-axis) and wrist (1756 counts per min; right Y-axis).

There were no advantages of a wrist-worn device over a hip-worn device for detecting SB. In fact, the resulting findings somewhat favor the hip position (see Table 4). To be specific, for the vertical axis, the specificity from the wrist cut-point (83.5%) was considerably lower than from the hip cut-point of 100CPM (92.5%), although similar sensitivity values were observed between the wrist (93.0%) and hip cut-point (93.7%). To illustrate this, a pattern of activity counts obtained from the vertical axis of the Actigraphs being placed at the hip and wrist for a 9-yr old female participant is presented on a single plot (See Figure 1). This participant performed 2 sedentary activities (sitting on a chair, playing a video game) and 10 non-sedentary activities in a random fashion for 5 min each (i.e. 60 min total). Activity counts either below or above the cut-point line were considered minutes spent sedentary or non-sedentary, respectively. For the 2 sedentary activities, the hip position correctly classified 8 of the 10 min as sedentary (Sensitivity = 93.7%) while the wrist correctly classified 9 of the 10 min (Sensitivity = 93.0%). However, for the 10 non-sedentary activities, the wrist position correctly classified only 43 of the 50 min as non-sedentary (Specificity = 83.5%) while the hip correctly classified 48 of the 50 min (Specificity = 93.0%).

A striking, but intuitive, finding herein was that most of the non-sedentary minutes misclassified as sedentary time were identified with ‘hand weight exercise’ for hip placement, and ‘stationary cycling at moderate pace’ and ‘stationary cycling at vigorous pace’ for wrist placement. As seen in Figure 1, 1 min of the total of 2 min of non-sedentary time misclassified as sedentary for hip placement was observed with ‘hand weight exercise’, with perfect classification for wrist placement for the same activity. In contrast, all the 5 min for the moderate activity of ‘stationary cycling at moderate pace’ were misclassified as sedentary time for the wrist, but were correctly classified as non-sedentary time for the hip. A potential explanation for this finding is that, when cycling, children move their hip to some extent for pedaling while their hands are nearly fixed onto the handles. When placed at the hip, the Actigraph can detect relatively more movements than when placed at the wrist, which may lead to higher values of specificity for the hip placement. In contrast, resistance exercise (e.g. hand weight exercise) involves considerably more arm movements (whereas the hip is nearly static), thus increasing the likelihood of activity being captured with the Actigraph being placed at the wrist. The three activities discussed are considered MVPAs based on their corresponding energy expenditure (i.e. ≥3.0 metabolic equivalents) [34]. However, these “quasi-MVPAs” largely accompany only either upper- or lower-body movement, so tend not to be precisely assessed with accelerometers that are worn at the hip. This has been regarded as one of the critical limitations of utilizing accelerometers in measuring youth physical activity [35]. Placing an Actigraph accelerometer on the wrist may overcome this limitation for resistance exercise. However, this practice in turn may increase chances of misclassifying cycling activities as sedentary, which would then result in overestimation of time spent in SB. Given that cycling is one of the most commonly observed youth physical activities, this overestimation may contribute considerably to the total estimated sedentary time in children. Pediatric public health researchers, policy makers, educators and/or surveillance studies (e.g. NHANES) should take this issue into account when assessing youth SB with an Actigraph accelerometer worn on a child's wrist.

To date, only a few studies [36], [37] have examined the utility of vector magnitude for assessing youth SB. A study by Hanggi et al. [36] has been performed to identify an optimal cut-point for vector magnitude for hip placement. A cut-point of 180CPM was identified therein as an optimal sedentary cut-point relative to indirect calorimetry in a sample of 49 children ages 10–15 yrs, which was far smaller than the cut-point of 554.3CPM identified in the present study. A potential explanation for this difference may be that our study included a substantially larger sample of children (i.e. 125 children vs. 49), used a wider variety of sedentary and physical activities (i.e. 24 activities vs. 8), and utilized a different criterion method (i.e. study protocol vs. indirect calorimetry). However, in another validation study by Crouter et al. [37], the 2-regression models (using vector magnitude) did not out-perform 1-regression models in estimating energy expenditure for sedentary activities. The two above-mentioned studies [36], [37] each evaluated data from hip-worn accelerometers, and no studies to date have reported on the use of vector magnitude for Actigraph accelerometers worn on the wrist.

A key factor that needs to be considered for research on youth SB is how SB is defined. For instance, SB is generally defined as activities that produce energy expenditure (EE) values minimally above one's resting metabolic rate [38]. SBhas been operationally defined as any activities that result in EE levels between 1.0MET and 1.5MET [38] but it can also be defined by posture: lying down, sitting, and screen-based activities. In the current study, we usedboth components in defining the criterion intensities of SB. To be specific, all the 5 sedentary activities included were performed while seated and with MET values between 1 and 1.5 based on the Compendium [29]. This ensured that the activities performed were indeed sedentary but a limitation is that the cut-points may or may not discriminate between sedentary and other light activities performed as part of daily living. For example, while SB can be approximated withlow activity counts (i.e. <100CPM), it is not possible to differentiate sitting (i.e. SB) from standing (i.e. light intensity). Therefore, it is possible that the cut-point method may misclassify standing as SB and result in overestimation of SB time.Inclinometer-based activity monitors (e.g. activPAL) have the ability to distinguish different body postures (i.e. sitting, standing, and walking). Recent Actigraph models (e.g. GT3X and GT3X+) equipped with an inclinometer offer potential for improved detection of SB but more work is needed [36]. Other approaches have been used to distinguish sedentary time from active time (e.g. 1- or 2-regression models) and these methods also enable MET-defined categorization of SB (i.e. Sedentary time estimated based on MET values between 1 and 1.5). These approaches offer promise for the future but the present findings provide guidance to ensure effective application of existing Actigraph data (collected at both the hip and wrist).

There are several strengths of this study. First of all, we directly compared outcomes from hip-worn Actigraphs to wrist-worn monitors while also presenting new empirically derived cut-points for both vertical axis and vector magnitude and for both hip and wrist placement. Internal validity of the study was strengthened by the use of direct observation as a reference criterion since the actual behaviors were directly recorded during the entire study protocol. External validity was strengthened by the use of a large and randomly selected set of commonly occurring sedentary activities. This design is unique among physical activity studies and it greatly enhances the ecological validity of the findings.

The findings of the present study still need to be interpreted with some caution. Only three light activities were included in this study. However, these light activities were similar to those performed in previous validation research [31]. In addition, this study may not represent true underlying characteristics of youth SB since the proposed activities were performed in a controlled research setting [35]. The protocol (and randomized set of activities) in the present study was intended to reflect real world settings, but the lab based environment is still artificial and may still limit the generalizability of the findings. However, the decision to use “simulated” free-living activities has been specifically recommended for cross-validation studies similar to the type conducted here [39]. Recent recommendations have also highlighted the potential of using data from cell phones and advanced analytical techniques (e.g. pattern recognition methods) to improve the detection of SB [40]. A previous study [41] showed that machine-learning techniquescan accurately classify sedentary activities as well as physical activities; however, the Actigraph was placed at the hip in that study [41]. Future research is clearly warranted to evaluate the potential of machine-learning techniques for detecting different types of SB (with Actigraphs or cell phones).A final limitation was that counts collected at 1 s epoch were reintegrated into 60 s epochs for data analysis. It is recommended to use an epoch length smaller than 5 s for youth physical activity research [42]. However, the methodology of reintegrating smaller epochs into larger epochs was supported by a previous study [43].

## Conclusion

The cut-point of 100CPM was supported as an optimal cut-point for vertical axis of the Actigraph placed at the hip. When assessing youth SB with an Actigraph placed on the wrist, the use of vertical axis cut-points developed for hip placement should be avoided. The optimal cut-points in our study were 554.3CPM for vector magnitude for hip placement, 1756CPM for vertical axis for wrist placement, and 3958.3CPM for vector magnitude for wrist placement. Additional work is needed to cross validate these proposed values under free-living environments. New technologies and analytical techniques should also be explored since they offer potential to overcome limitations of using cut-points. However, there is considerable work still being conducted with Actigraph monitors and the proposed values would facilitate standardization in approaches for evaluating SB in youth.
